# Insights into *Vibrio parahaemolyticus* CHN25 Response to Artificial Gastric Fluid Stress by Transcriptomic Analysis

**DOI:** 10.3390/ijms151222539

**Published:** 2014-12-05

**Authors:** Xuejiao Sun, Taigang Liu, Xu Peng, Lanming Chen

**Affiliations:** 1 Key Laboratory of Quality and Safety Risk Assessment for Aquatic Products on Storage and Preservation (Shanghai), China Ministry of Agriculture; College of Food Science and Technology, Shanghai Ocean University, 999 Hu Cheng Huan Road, Shanghai 201306, China; E-Mail: m120250546@st.shou.edu.cn; 2 College of Information Technology, Shanghai Ocean University, 999 Hu Cheng Huan Road, Shanghai 201306, China; E-Mail: tgliu@shou.edu.cn; 3 Archaea Centre, Department of Biology, University of Copenhagen, Ole Maaløes Vej 5, DK2200 Copenhagen N, Denmark; E-Mail: peng@bio.ku.dk

**Keywords:** *Vibrio parahaemolyticus*, acid stress, transcriptome, gene expression, growth phase

## Abstract

*Vibrio parahaemolyticus* is the causative agent of food-borne gastroenteritis disease. Once consumed, human acid gastric fluid is perhaps one of the most important environmental stresses imposed on the bacterium. Herein, for the first time, we investigated *Vibrio parahaemolyticus* CHN25 response to artificial gastric fluid (AGF) stress by transcriptomic analysis. The bacterium at logarithmic growth phase (LGP) displayed lower survival rates than that at stationary growth phase (SGP) under a sub-lethal acid condition (pH 4.9). Transcriptome data revealed that 11.6% of the expressed genes in *Vibrio parahaemolyticus* CHN25 was up-regulated in LGP cells after exposed to AGF (pH 4.9) for 30 min, including those involved in sugar transport, nitrogen metabolism, energy production and protein biosynthesis, whereas 14.0% of the genes was down-regulated, such as ATP-binding cassette (ABC) transporter and flagellar biosynthesis genes. In contrast, the AGF stress only elicited 3.4% of the genes from SGP cells, the majority of which were attenuated in expression. Moreover, the number of expressed regulator genes was also substantially reduced in SGP cells. Comparison of transcriptome profiles further revealed forty-one growth-phase independent genes in the AGF stress, however, half of which displayed distinct expression features between the two growth phases. *Vibrio parahaemolyticus* seemed to have evolved a number of molecular strategies for coping with the acid stress. The data here will facilitate future studies for environmental stresses and pathogenicity of the leading seafood-borne pathogen worldwide.

## 1. Introduction

*Vibrio parahaemolyticus*, autochthonous to estuarine, marine, and coastal environments worldwide, is the causative agent of food-borne gastroenteritis disease and even death [1]. *V. parahaemolyticus* was first identified in 1950 in Osaka, Japan, where an outbreak of acute gastroenteritis following the consumption of semidried juvenile sardines sickened 272 and killed 20 individuals [2]. To date, more than eighty *V. parahaemolyticus* serotypes have been described on the basis of the somatic (O) and capsular (K) antigens [1]. Epidemic *V. parahaemolyticus* O3:K6 emerged in Calcutta, India in 1996 [3], was subsequently isolated in many Asian countries, and recently reported in Europe, Africa and America [1,4], arguing a pandemic of *V. parahaemolyticus* worldwide.

*V. parahaemolyticus* is a Gram-negative bacterium that is able to grow at pH 5–11, 1%–7% NaCl, 22–42 °C [5,6]. Once consumed with raw, undercooked or mishandled seafood, *V. parahaemolyticus* is challenged with the extremely low pH environment in the human stomach (pH of the human stomach normally ranges from 1–3 but can rise above 6.0 after food consumption) [7,8], before reaching the human gastrointestinal tract where it elicits gastroenteritis [9]. The molecular mechanisms of acid stress response in some Gram positive and Gram-negative bacteria (e.g., *Escherichia coli*, *Salmonella enterica*) have been reported, such as the pumping out of protons, production of ammonia and proton-consuming decarboxylation reactions, as well as modifications of the lipid content in the membrane (for a review, see [10]).

To date, the general stress response of *Vibrionaceae*-related bacteria under detrimental acid conditions remains largely unknown, despite their great significance in human health and economy in aquaculture industry. Some studies have revealed that *Vibrios* have a similar lysine-decarboxylation pathway in response to acid stress as *E. coli*, which consists of a lysine decarboxylase (CadA) and a lysine/cadaverine antiporter (CadB). The *cadA* and *cadB* genes were transcribed at low constitutive levels in an acid-independent manner and induced during infection and acid tolerance in *Vibrio cholerae* [11], and the genes were activated sequentially by two transcriptional regulators AphB and CadC of *Vibrio vulnifus* in acid stress [12]. Short preadaptation to a 6% salt concentration increased survival of the wild-type strain but not that of a *cadA* mutant of *V. parahaemolyticus* under lethal acid conditions [13]. Previous research on specific genes also revealed a few regulatory proteins (e.g., ToxRS and OmpU) involved in *V. parahaemolyticus* response to acid, bile salts, and sodium dodecyl sulfate stresses (e.g., [14]). In this study, for the first time, we investigated global-level gene expression profiles of *V. parahaemolyticus* CHN25 in response to artificial gastric fluid (AGF) stress by using full-genome microarray analysis. The information will facilitate our better understanding of molecular mechanisms underlying environmental stresses and pathogenicity of the leading seafood-borne pathogen worldwide.

## 2. Results and Discussion

### 2.1. Survival of V. parahaemolyticus CHN25 under Acid pH Conditions

**Figure 1:**
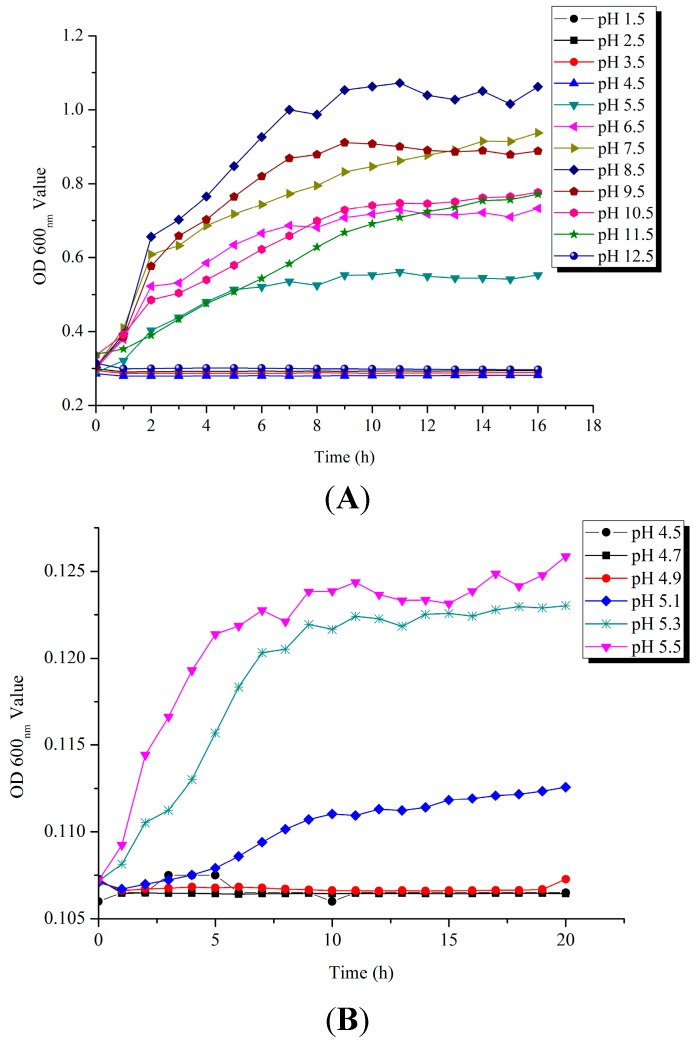
Survival of *V. parahaemolyticus* CHN25 under different pH conditions. The bacterium was grown in TSB liquid medium at pH 1.5–12.5 (**A**) and pH 4.5–5.5 (**B**), 37 °C, and growth curves were determined using a BioScreener.

To gain an insight into the *V. parahaemolyticus* CHN25 tolerance to acid conditions, we determined growth curves of the bacterium, recently isolated and identified by Song *et al.* [15], in Tryptic Soy Broth (TSB) with the pH range of 1.5–12.5 at 37 °C. As illustrated in Figure 1A, *V. parahaemolyticus* CHN25 grows at pH 5.5–11.5, optimally at pH 8.5, demonstrating it is a moderately basophilic bacterium, consistent with previous studies (e.g., [5]). No cell growth was observed under more acidic conditions with pH values lower than 4.5. More detailed tests on the pH range between 4.5 and 5.5 revealed that *V. parahaemolyticus* CHN25 was able to grow at pH 5.0, but not at pH ≤ 4.9 (Figure 1B), suggesting the latter being a sub-lethal pH condition for *V. parahaemolyticus* CHN25.

### 2.2. Tolerance of V. parahaemolyticus CHN25 at Logarithmic Growth Phase (LGP) and Stationary Growth Phase (SGP) to the AGF (Artificial Gastric Fluid) Stress

**Figure 2:**
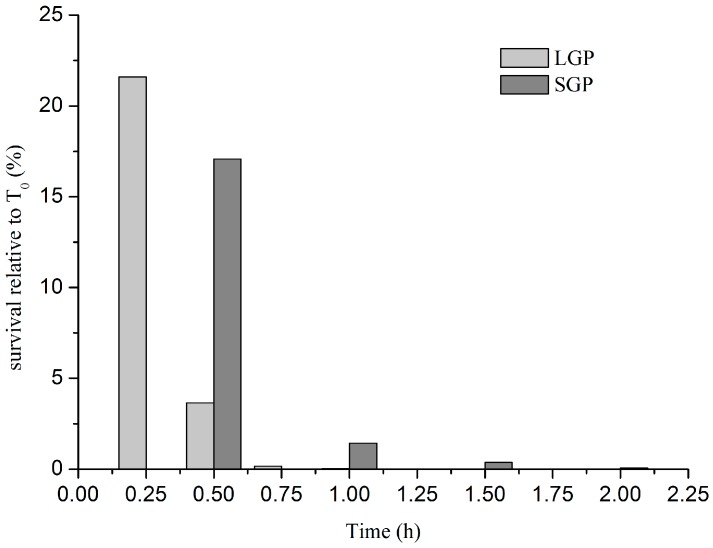
Tolerance of *V. parahaemolyticus* CHN25 at LGP (logarithmic growth phase) and SGP (stationary growth phase) to the AGF (artificial gastric fluid) (pH 4.9) stress.

To investigate the possible effects of the human acidic stomach environment *in vivo* on *V. parahaemolyticus* CHN25 survival, we utilized the AGF (pH 4.9) to treat the bacterium *in vitro* grown to LGP and SGP in TSB (pH 8.5) at 37 °C, respectively. As shown in Figure 2, *V. parahaemolyticus* CHN25 cells at LGP displayed relatively lower survival rates when compared to the bacterial cells at SGP. Treating the LGP cells for 15 min resulted in a significantly decreased survival rate (21.6%), and further elevating exposure time (≥30 min) yielded a steep reduction in the survival (≤3.7%). For the SGP cells, the relative survival rate was 17.0% after exposed to AGF for 30 min, which was 4.6-fold higher than that for LGP cells. Nevertheless, the SGP cells also showed considerable loss in culturability after 30 min exposure to AGF. Thus, we extracted total RNA of the samples at both growth phases in TSB after treated for 30 min with AGF (pH 4.9) for the further transcriptomic analysis (see below). The samples cultured under the same condition without the AGF treatment were used as a control, respectively.

### 2.3. Transcriptome Profiles of V. parahaemolyticus CHN25 in the Response to the AGF Stress

We determined the global-level gene expression profiles of *V. parahaemolyticus* CHN25 after the AGF treatment by using full-genome microarray chips (see the Experimental Section). This analysis revealed a considerable number of differentially expressed genes involved in the response to acid stress in the bacterium. A total of 1210 genes were significantly changed when *V. parahaemolyticus* CHN25 grown to LGP in the AGF stress, which represented approximately 25.6% of the expressed genes in the bacterium. Of these, a total of 547 genes showed higher transcriptional levels (change ≥ 2.0-fold), whereas the expression of a total of 663 genes were down-regulated (change ≤ 0.5-fold). All the genes were grouped into ninety four gene functional catalogues identified in the Kyoto Encyclopedia of Genes and Genomes (KEGG) database (data not shown). When *V. parahaemolyticus* CHN25 grown to SGP, the AGF stress only elicited 160 differentially expressed genes, accounting for 3.4% of the expressed genes in the bacterium, which consisted of 52 up-regulated and 108 down-regulated genes falling into twenty two gene functional catalogues (data not shown). A complete list of the differentially expressed genes at both growth phases is available in NCBI Gene Expression Omnibus under the accession number GSE63167, of which 10.9% were annotated as hypothetical proteins with currently unknown functions in the public databases. To validate the transcriptome data, we chose eleven representative genes for quantitative real-time reverse transcription PCR (qRT-PCR) analysis. The resulting data were correlated with those yielded from the transcriptomic analysis (Table S1).

Consistent with previous studies (e.g., [16]), the majority of the expressed genes remained unaltered in SGP cells in the AGF stress, in which substrate metabolism, energy production, and cell division were turned down. Nevertheless, the transcriptome profile at this growth phase indeed provided a comparative mode to investigate growth-phase independent acid stress response in *V. parahaemolyticus* CHN25. Comparison of the transcriptome data revealed forty-one differentially expressed genes that were synchronously elicited from both LGP and SGP cells in the AGF stress, however, approximately half of which coded for hypothetical proteins. Of these, interestingly, almost half displayed different expression features between the two growth phases, e.g., the genes encoding the fructose-specific Enzyme IIABC subunits and prophage-associated proteins (see below). Overall, our data highlighted characteristic and distinct gene expression patterns of *V. parahaemolyticus* CHN25 with considerable variation over growth phases in the AGF stress.

### 2.4. Major Metabolic Pathways Involved in the Response of V. parahaemolyticus CHN25 to the AGF Stress

#### 2.4.1. Major Metabolic Pathways Involved in *V. parahaemolyticus* CHN25 Cells at LGP in the AGF Stress

Based on gene set enrichment analysis (GSEA) of the transcriptome data against the KEGG database, nine significantly affected metabolic pathways with enrichment test p value below 0.05 were identified in LGP cells after exposed to the AGF stress (Table 1). They included the phosphotransferase system (PTS); galactose, nitrogen, fructose and mannose, and pyruvate metabolisms; ribosome and aminoacyl-tRNA biosynthesis; glycolysis/gluconeogenesis; and oxidative phosphorylation. The significantly changed metabolic pathways have also been reported in some other bacteria under acid conditions (e.g., [16,17,18,19,20]).

The phosphoenolpyruvate-dependent PTS is known as a major sugar transport multicomponent system in bacteria, by which many sugars are transported into bacteria, concomitantly phosphorylated, and then fed into glycolysis [21]. In this study, thirteen genes in the PTS were significantly up-regulated in LGP cells in the AGF stress, which coded for glucose-, fructose-, mannitol-, cellobiose-, ascorbate-, and *N*-acetylglucosamine-specific enzyme II components. One of these genes, VPA1424 encoding fructose-specific enzyme II ABC subunits, was strongly up-regulated for 27.97-fold in mRNA level, suggesting extremely active transport and utilization of the fructose in the AGF stress. In addition, interestingly, two genes encoding glucose-specific enzyme II BC subunits were identified in *V. parahaemolyticus* CHN25, one of which (VP2046) was induced with a minor increase of 2.62-fold, whereas the other (VPA1667) strikingly displayed a 25-fold decrease in expression, implying possible unknown regulation mechanisms underlying the transport of the key sugar in central carbohydrate metabolism in the AGF stress.

**Table 1:** Major metabolic pathways and cellular functions involved in the response of *V. parahaemolyticus* CHN25 at LGP and SGP to the AGF (pH 4.9) stress for 30 min.

Metabolic Pathway/Cellular Function	Locus/Gene in *V. parahaemolyticus*		Fold Change	Description of Encoded Protein
	RIMD2210633	CHN25		
*LGP cells*				
Phosphotransferase system (PTS)	VPA1667	Chn25A_1555	0.0384	Glucose-specific IIBC component
	VPA1424	Chn25A_1312	27.9696	Fructose-specific IIABC component
	VPA1422	Chn25A_1310	2.2688	Nitrogen regulatory IIA component
	VPA1421	Chn25A_1309	2.3425	Fructose-specific IIB component
	VPA1420	Chn25A_1308	2.8724	Fructose-specific IIABC component
	VPA0501	Chn25A_1196	2.6299	Mannitol-specific enzyme II component
	VPA0500	Chn25A_1197	2.6362	Mannitol-specific enzyme II component
	VPA0297	Chn25A_0302	8.029	fructose-specific IIBC component
	VPA0298	Chn25A_0303	2.8846	Fructose-specific IIA component
	VPA0231	Chn25A_0232	2.0079	Phosphotransferase enzyme II, A component
	VPA0230	Chn25A_0231	5.501	Putative sugar phosphotransferase component II B
	VP2637	Chn25_2566	2.2518	Cellobiose-specific IIB component
	VP2636	Chn25_2565	2.6756	Cellobiose-specific IIC component
	VP2046	Chn25_1932	2.6196	Glucose-specific IIBC components
	VP0831	Chn25_0826	4.8477	*N*-acetylglucosamine-specific IIABC component
	VP0370	Chn25_0356	2.7113	Mannitol-specific IIABC component
	VPA0229 (*ulaA*)	Chn25A_0230	6.4614	Ascorbate-specific enzyme IIC
	VP0366	Chn25_0352	3.2996	Putative PTS, enzyme I
Galactose metabolism	VPA0879	Chn25A_0828	0.3848	UDP-glucose 4-epimerase
	VP2400	Chn25_2264	4.517	UDP-glucose 4-epimerase
	VP2400	Chn25A_1071	3.5838	UDP-glucose 4-epimerase
	VP2399	Chn25A_1070	4.4699	Galactose-1-phosphate uridylyltransferase
	VP2398	Chn25_2262	3.8991	Galactokinase
	VP2398	Chn25A_1069	2.497	Galactokinase
	VP2077	Chn25_1963	2.0087	Maltodextrin glucosidase
Galactose metabolism	VP0839	Chn25_0834	2.4836	Phosphoglucomutase
	VP2403 (*ebgA*)	Chn25_2266	2.4115	Cryptic beta-d-galactosidase subunit alpha
	VP2404 (*ebgC*)	Chn25_2267	2.9965	Cryptic beta-d-galactosidase subunit beta
	VP2397 (*galM*)	Chn25_2261	2.1978	Aldose 1-epimerase
	VP2855 (*pfkA*)	Chn25_2776	0.3195	6-phosphofructokinase
Ribosome biosynthesis	VP2772	Chn25_2698	3.0673	30S ribosomal protein S7
	VP1210	Chn25_1217	2.6329	50S ribosomal protein L25
	VP2925 (*rplA*)	Chn25_2832	2.3329	50S ribosomal protein L1
	VP2926 (*rplK*)	Chn25_2833	2.1866	50S ribosomal protein L11
	VP2923 (*rplL*)	Chn25_2830	2.7378	50S ribosomal protein L7/L12
	VP0264 (*rplP*)	Chn25_0255	2.2242	50S ribosomal protein L16
	VP1282 (*rplT*)	Chn25_1289	6.5113	50S ribosomal protein L20
	VP0262 (*rplV*)	Chn25_0253	2.5181	50S ribosomal protein L22
	VP0259 (*rplW*)	Chn25_0250	2.2719	50S ribosomal protein L23
	VP0329 (*rpmA*)	Chn25_0317	5.5738	50S ribosomal protein L27
	VP0185 (*rpmB*)	Chn25_0181	2.7392	50S ribosomal protein L28
	VP0265 (*rpmC*)	Chn25_0256	2.5323	50S ribosomal protein L29
	VP0255 (*rpmE*)	Chn25_0246	5.0193	50S ribosomal protein L31
	VP0186 (*rpmG*)	Chn25_0182	7.2192	50S ribosomal protein L33
	VP2030 (*rpsA*)	Chn25_1918	3.9866	30S ribosomal protein S1
	VP0263 (*rpsC*)	Chn25_0254	2.6074	30S ribosomal protein S3
	VP2740 (*rpsF*)	Chn25_2668	2.818	30S ribosomal protein S6
	VP0439 (*rpsI*)	Chn25_0398	2.1455	30S ribosomal protein S9
	VP2453 (*rpsO*)	Chn25_2391	3.7221	30S ribosomal protein S15
	VP0266	Chn25_0257	3.3225	Ribosomal protein S17
	VP0531 (*rpsT*)	Chn25_0481	6.127	30S ribosomal protein S20
Fructose and mannose metabolism	VPA1425	Chn25A_1313	23.9303	Mannose-6-phosphate isomerase
	VP2599	Chn25_2528	0.2922	Fructose-bisphosphate aldolase
	VP2488	Chn25_2426	4.6724	Putative phosphoglucomutase/phosphomannomutase
Amino sugar and nucleotide sugar metabolism	VP0543	Chn25_0493	2.2962	*N*-acetylmuramic acid-6-phosphate etherase
	VPA0833 (*glgC*)	Chn25A_0780	0.4431	Glucose-1-phosphate adenylyltransferase
	VP1023 (*glgC*)	Chn25_1047	2.336	Glucose-1-phosphate adenylyltransferase
	VP0829 (*nagA*)	Chn25_0825	2.027	*N*-acetylglucosamine-6-phosphate deacetylase
	VPA0038 (*nagB*)	Chn25A_0033	2.5647	Glucosamine-6-phosphate deaminase
Glycolysis/gluconeogenesis	VPA0566	Chn25A_1136	5.8196	Alcohol dehydrogenase
	VPA0180	Chn25A_0182	2.2002	Phospho-beta-glucosidase B
	VP2157	Chn25_2026	4.6439	Glyceraldehyde-3-phosphate dehydrogenase
Aminoacyl-tRNA biosynthesis	VP2470	Chn25_2407	2.0793	Tyrosyl-tRNA synthetase
	VP1280	Chn25_1286	0.4891	Threonyl-tRNA synthetase
	VP2548 (*alaS*)	Chn25_2482	2.6177	Alanyl-tRNA synthetase
	VP0861 (*argS*)	Chn25_0854	2.7778	Arginyl-tRNA synthetase
	VP1150 (*cysS*)	Chn25_1159	2.0297	Cysteinyl-tRNA synthetase
	VP0021 (*glyS*)	Chn25_0010	3.0031	Glycyl-tRNA synthetase subunit beta
	VP0534 (*ileS*)	Chn25_0484	2.3183	Isoleucyl-tRNA synthetase
	VP0727 (*leuS*)	Chn25_0685	2.1816	Leucyl-tRNA synthetase
	VP2069 (*metG*)	Chn25_1955	2.2087	Methionyl-tRNA synthetase
	VP1291 (*pheT*)	Chn25_1298	5.4809	Phenylalanyl-tRNA synthetase subunit beta
	VP2646 (*valS*)	Chn25_2575	2.077	Valyl-tRNA synthetase
Pyruvate metabolism	VPA1567	Chn25A_1456	2.1699	Putative pyruvate formate lyase
	VPA1123	Chn25A_0560	0.4528	Putative acyl-CoA thiolase
	VPA0823	Chn25A_0771	2.0537	Pyruvate kinase
	VPA0646	Chn25A_1010	0.3383	Putative pyruvate dehydrogenase E1 component, beta subunit
Pyruvate metabolism	VPA0620	Chn25A_1034	0.4695	Putative acyl-CoA thiolase
	VPA0611	Chn25A_1091	0.3706	Acetate kinase
	VPA0372	Chn25A_0367	3.9537	Phosphoenolpyruvate synthase
	VPA0144	Chn25A_0145	0.4364	d-lactate dehydrogenase
	VP2881	Chn25_2801	3.0293	Acetyl-CoA carboxylase, biotin carboxylase subunit
	VP2878	Chn25_2798	2.514	Acetyl-CoA synthetase
	VP2545	Chn25_2479	2.1607	Oxaloacetate decarboxylase subunit gamma
	VP2517	Chn25_2451	3.4256	Dihydrolipoamide dehydrogenase
	VP2039	Chn25_1927	2.6156	Pyruvate kinase II
	VP1627	Chn25_1620	3.6175	Acylphosphatase
	VP1258	Chn25_1264	2.6731	Malate dehydrogenase
	VP0325	Chn25_0313	3.5504	Malate dehydrogenase
	VP2519 (*aceE*)	Chn25_2453	2.132	Pyruvate dehydrogenase subunit E1
	VPA1499 (*lldD*)	Chn25A_1389	8.5267	l-lactate dehydrogenase
Oxidative phosphorylation	VPA0631	Chn25A_1023	0.464	Putative protoheme IX farnesyltransferase
	VPA0544	Chn25A_1156	0.4221	Protoheme IX farnesyltransferase
	VP2841	Chn25_2763	2.102	Fumarate reductase iron-sulfur subunit
	VPA0539	Chn25A_1161	0.2437	Cytochrome c oxidase, subunit III
	VP1543	Chn25_1521	2.2303	Cytochrome c oxidase, subunit CcoO
	VP1541	Chn25_1519	2.2683	Cytochrome c oxidase, subunit CcoP
	VP1054	Chn25_1074	2.0957	Cytochrome d ubiquinol oxidase, subunit II
	VP1053	Chn25_1073	2.3358	Cytochrome d ubiquinol oxidase, subunit I
	VPA0628	Chn25A_1026	2.0087	Cytochrome o ubiquinol oxidase, subunit I
	VP1165	Chn25_1174	2.4205	Putative manganese-dependent inorganic pyrophosphatase
	VP0443	Chn25_0401	2.4995	Ubiquinol-cytochrome c reductase, cytochrome c1
	VP0442	Chn25_0400	2.0404	Ubiquinol-cytochrome c reductase, cytochrome b
Oxidative phosphorylation	VP3076	Chn25_2976	0.4698	F0F1 ATP synthase subunit I
	VP3068 (*atpC*)	Chn25_2968	3.0803	F0F1 ATP synthase subunit epsilon
	VP0844	Chn25_0839	2.0288	Succinate dehydrogenase, hydrophobic membrane anchor protein
	VP0843 (*sdhC*)	Chn25_0838	2.1718	Succinate dehydrogenase cytochrome b556 large membrane subunit
Ciliary or bacterial-type flagellar motility	VP0772 (*flgA*)	Chn25_0767	0.2303	Flagellar basal body P-ring biosynthesis protein FlgA
	VP2235 (*flhA*)	Chn25_2102	0.4555	Flagellar biosynthesis protein FlhA
	VP2236 (*flhB*)	Chn25_2103	0.463	Flagellar biosynthesis protein FlhB
	VP2255	Chn25_2122	0.436	Polar flagellar rod protein FlaI
	VP2256 (*fliD*)	Chn25_2123	0.4776	Flagellar capping protein
	VP2257	Chn25_2124	2.0357	Flagellar protein FlaG
	VP2261	Chn25_2127	0.2235	Flagellin
	VPA0263	Chn25A_0264	0.3335	Flagellar basal body P-ring biosynthesis protein
Polyamine transport	VP1332	Chn25_1334	0.4055	Binding protein component of ABC transporter
	VP1336	Chn25_1337	0.3839	ABC transporter ATP-binding protein
	VP1337	Chn25_1338	0.419	Putative permease of ABC transporter
	VP1338	Chn25_1339	0.371	ABC transporter permease
d-ribose transport	VPA1087	Chn25A_0593	12.5376	d-ribose pyranase
	VPA1086	Chn25A_0594	14.963	d-ribose transporter ATP binding protein
	VPA1086 (*rbsC*)	Chn25A_0595	8.678	Ribose ABC transporter permease protein
	VPA1084	Chn25A_0596	7.7049	d-ribose transporter subunit RbsB
Maltose transport	VPA1399 (*malG*)	Chn25A_1076	2.0352	Maltose transporter permease
	VPA1400 (*malF*)	Chn25A_1077	2.8009	Maltose transporter membrane protein
	VPA1401(*malE*)	Chn25A_1078	3.0226	Maltose ABC transporter periplasmic protein
	VPA1402	Chn25A_1079	2.561	Maltose/maltodextrin transporter ATP-binding protein
	VPA1644 (*lamB*)	Chn25A_1532	3.35	Maltoporin
*SGP cells*				
Pyrimidine and purine metabolism	VPA1243	Chn25A_0442	0.491	Cytosine deaminase
	VP0524 (*thyA*)	Chn25_0476	2.1909	Thymidylate synthase
	VP1760	Chn25_1387	2.1851	Putative adenylate cyclase
	VPA1159	Chn25A_0527	0.4775	Guanosine 5'-monophosphate oxidoreductase
	VPA0855	Chn25A_0801	0.3912	Putative 5'-nucleotidase
	VPA0074	Chn25A_0069	2.3652	Putative DNA polymerase III, epsilon subunit
	VP2303 (*dnaE*)	Chn25_2169	0.4586	DNA polymerase III subunit alpha
Iron ion transport	VP2491	Chn25_2429	0.4159	Iron (III) ABC transporter, periplasmic Iron-compound-binding protein
	VPA0310	Chn25A_0316	2.1424	Hypothetical protein
PTS	VP2674	Chn25_2602	2.2237	Phosphocarrier protein NPr
	VPA0297	Chn25A_0302	0.4786	PTS system, fructose-specific IIBC component
	VPA0298	Chn25A_0303	0.4004	PTS system, fructose-specific IIA component
	VPA1424	Chn25A_1312	0.3946	PTS system, fructose-specific IIABC component
Quaternary ammonium group transport	VPA1111	Chn25A_0571	0.453	Putative glycine betaine-binding ABC transporter
Aromatic compound catabolic process	VP0240	Chn25_0231	2.2949	Putative 5-carboxymethyl-2-hydroxymuconate delta isomerase
Glycine betaine biosynthetic process from choline	VPA1114	Chn25A_0568	0.4435	Transcriptional regulator BetI
	VPA1112	Chn25A_0570	0.3776	Choline dehydrogenase
Pilus	VPA0725	Chn25A_0670	3.0408	Putative TadB
ATP binding	VPA0380	Chn25A_0375	0.481	Hypothetical protein
	VPA1302	Chn25A_0400	0.2424	Hypothetical protein
Outer membrane-bounded periplasmic space and nitrite reductase activity	VP1928	Chn25_1816	2.0205	Cytochrome c nitrite reductase pentaheme subunit
Cytolysis	VP3048	Chn25_2947	2.0825	Putative hemolysin III
Betaine-aldehyde dehydrogenase activity	VPA1113	Chn25A_0569	0.3189	Betaine aldehyde dehydrogenase
Phosphoglycerate transport	VPA0825	Chn25A_0773	0.3614	Putative phosphoglycerate transport regulatory protein PgtC
NADPH dehydrogenase activity	VPA0465	Chn25A_1233	2.0487	Putative NAD(P)H oxidoreductase
Regulation of DNA repair	VP2945	Chn25_2852	2.205	LexA repressor
3-isopropylmalate dehydratase complex and 3-isopropylmalate dehydratase activity	VP0343	Chn25_0331	2.0142	Isopropylmalate isomerase large subunit
Triglyceride lipase activity	VP1181	Chn25_1190	2.2305	lactonizing lipase
Lactoylglutathione lyase activity	VP2166	Chn25_2034	0.3883	Putative lactoylglutathione lyase
Anaerobic electron transport chain and nitrogen compound metabolic process	VP1928	Chn25_1816	2.0205	Cytochrome c nitrite reductase pentaheme subunit
Alkaline phosphatase activity	VP2163	Chn25_2032	0.4035	Alkaline phosphatase
Zinc ion transmembrane transporter activity	VPA1287	Chn25A_0416	0.2585	Putative transporter
Transmembrane transport	VP1359	Chn25_1745	2.0727	Hypothetical protein

The Leloir pathway for the catabolism of d-galactose was positively affected in the AGF stress, which produces UDP-glucose, an important building block for glycogen biosynthesis. Consistent with previous research (e.g., [16]), the genes encoding the pathway components were significantly up-regulated in the AGF stress (Table 1).

Approximately half of the genes linked to nitrogen metabolism were significantly changed by the AGF stress, the majority of which showed higher transcriptional levels. Interestingly, four enzymes: 2-nitropropane dioxygenase (VPA0296) catalyzing nitroalkane to nitrite; NrfBD (VP0987) and NrfAH (VP1989) involved in the conversion of nitrite to ammonia in dissimilar nitrate reduction; and nitrite reductase large subunit (VPA0987) involved in the catalysis of nitrite to ammonia, were significantly up-regulated. Moreover, the gene encoding a glutamate dehydrogenase (VP1602) that catalyzes l-glutamate to ammonia was up-regulated as well, suggesting possibly increased amount of ammonia in LGP cells that likely combined with intracellular protons to yield the ammonium ion and alkalized intracellular environment in the AGF stress [10]. On the other hand, the gene (VP0483) involved in the conversions of ammonia to l-glutamine and to l-glutamate was significantly down-regulated, implying perhaps attenuated ammonia utilization in the AGF stress. In addition, a carbonic anhydrase (VP2514) that converts carbon dioxide to HCO_3_^−^ was notably down-regulated (5.27-fold), suggesting the repressed production of electrically negative acid ions in LGP cells, which may facilitate to maintain intracellular pH homeostasis in the AGF stress. To our knowledge, no linking to acid stress of the latter two genes has been described previously.

Bacterial ribosome consists of two major subunits, each of which is composed of a variety of proteins. Inconsistent with some previous studies showing down-regulated ribosomal genes under acidic conditions (e.g., [20]), in this study, twelve genes encoding the large 50S ribosomal subunit component were up-regulated (2.19–7.22-fold) in LGP cells after exposed to the AGF stress. Similarly, seven components of the small 30S subunit were also up-regulated in expression (2.15–6.13-fold) (Table 1). Despite a highly conserved translational machinery with invariable rRNA and protein components, the formation of distinct ribosomal subpopulations has been reported in bacteria when encountered adverse conditions, e.g., the S21, L2 and L20 subpopulations at pH 4.5 urea condition in *E. coli* [22,23]. In this study, L20 and some other components (S20, L27, L31 and L33) were highly up-regulated for more than 5.0-fold in the AGF stress. It will be interesting to elucidate biological significance of the enhanced ribosome synthesis and possible ribosomal subpopulations in *V. parahaemolyticus* CHN25 to the AGF stress in future research.

In the fructose and mannose metabolisms, two enzymes, bifunctional phosphomannomutase/phosphoglucomutase (VPA2488) and mannose-6-phosphate isomerase (VPA1425) that functioned in the conversions of d-mannose-1 phosphate to β-d-fructose-6 phosphate, were up-regulated in LGP cells. Interestingly, the latter exhibited a 23.93-fold enhanced expression, which reinforced the extremely active fructose metabolism in the AGF stress. All the differentially expressed genes in aminoacyl-tRNA biosynthesis were also up-regulated (2.01–5.48-fold), except the gene (VP1289) with a minor decrease (Table 1).

In the glycolysis/gluconeogenesis, the *pfkA* (VP2855) gene encoding a 6-phosphofructokinase that catalyzes the second rate-limiting reaction in glycolysis was down-regulated. The following reaction catalyzed by a fructose-bisphosphate aldolase (VP2599) was repressed as well. In contrast, three genes (VP2157, VPA0823, VP2039) in the pathway were up-regulated, the latter two of which coded for pyruvate kinases catalyzing the last rate-limiting reaction in glycolysis, suggesting possibly active pyruvate metabolism in LGP cells in the AGF stress.

Approximately 20 genes linked to the pyruvate metabolism were significantly elicited from LGP cells by the AGF stress. Of these, five genes were down-regulated, and the others were up-regulated. Interestingly, the *lldD* (VPA1499) gene encoding an l-lactate dehydrogenenase displayed an increase of 8.53-fold in expression, which degrades l-lactate to pyruvate. Moreover, the conversion of malate to pyruvate catalyzed by a malate oxidoreductase (VP1258) was also enhanced. The increased amount of pyruvate was actively metabolized by a phosphoenolpyruvate synthase (VPA0372) to produce phosphoenolpyruvic acid that enters into glycolysis. Similarly, the acetyl-CoA synthetase (VP2878) that catalyzes acetate to acetyl-CoA, an efficient substrate for tricarboxylic acid (TCA) cycle, was also up-regulated. These data suggested active l-lactate, malate and acetate metabolisms in LGP cells in the acid stress, consistent with previous research (e.g., [17]).

In oxidative phosphorylation, strikingly, the most enhanced was the *atpC* gene (VP3068), encoding ε subunit of a multisubunit F0F1-ATPase, which synthesizes ATP aerobically, as a result of protons passing into the cell, or hydrolyzes ATP for the expulsion of protons from cytoplasm anaerobically [24]. The up-regulated F0F1-ATPase gene operon has been reported in some other bacteria in bile and acid stresses (e.g., [20]). In this study, the enhanced expression of ε subunit of the F0F1-ATPase, which is located in a common central stalk linking the F0 and F1 rotary motors [25], suggested perhaps active pumping of excessive protons from LGP cells after exposed to a sub-lethal acid condition (pH 4.9).

#### 2.4.2. Major Metabolic Pathways Involved in *V. parahaemolyticus* CHN25 Cells at SGP in the AGF Stress

Based on the GESA-KEEG analysis, only four metabolic pathways, including pyrimidine, purine, as well as fructose and mannose metabolisms and the PTS, were identified to be significantly changed in SGP cells after exposed to the AGF stress (*p* < 0.05) (Table 1). Distinct from LGP cells, the genes encoding fructose-specific Enzyme IIA subunit (VPA0298) and fructose-specific enzyme II ABC subunits (VPA1424) involved in the PTS and fructose and mannose metabolisms were significantly down-regulated, indicating possibly reduced fructose transport in SGP cells in the AGF stress. In addition, consistent with some previous studies (e.g., [17]), the majority of the differentially expressed genes involved in pyrimidine and purine metabolisms were also down-regulated, e.g., DNA polymerase III α and ε subunits (VP2303, VPA0074), suggesting likely reduced DNA synthesis in SGP cells in the AGF stress.

### 2.5. Other Altered Biological Functions in V. parahaemolyticus CHN25 in the Response to the AGF Stress

The GSEA of the differentially expressed genes against the Go Ontology (GO) database revealed several significantly affected biological functions (*p* < 0.05) in LGP cells in the AGF stress (Table 1). Of these, the d-ribose and maltose/maltodextrin transport systems were significantly enhanced. The ATP-binding cassette (ABC) transporters are known as molecular pumps that harness the chemical energy of ATP hydrolysis to translocate solutes across the membrane [26]. Significantly changed ABC transporters have been reported in some other bacteria after acid shock (e.g., [17]). In this study, expression of the *rbsABCD* operon encoding d-ribose transporter components was strongly enhanced for 7.70–14.96-fold. Enhanced expression of several genes involved in sugar transport and utilization (e.g., ribose) has also been observed in *Lactobacillus plantarum* in the gastrointestinal tract of mice [27]. Similarly, five genes in the maltose/maltodextrin transport system were also up-regulated (Table 1). These data suggested active d-ribose and maltose/maltodextrin ABC transport systems in the AGF stress.

In contrast, two biological functions were significantly repressed in LGP cells in the AGF stress. One of these was the flagellar biosynthesis and motility, in which all the eight differently expressed genes were notably down-regulated (2.08–4.55-fold), except the *flaG* gene encoding a distal rod protein with a minor increase in mRNA level. They included the *flhA*, *flhB*, *flicC*, *flicD*, *flgA*, VPA0263 and VP2255. Flagellum motility is generally thought to be extremely energy consumptive under detrimental conditions. Albeit previous studies gave different expression characteristics of the genes involved in flagellar biosynthesis and motility under acidic conditions (e.g., [18,28,29,30]), our data strongly suggested the reduced biosynthesis of the flagellum structure and or flagellum motibity in *V. parahaemolyticus* CHN25 cells at LGP in the AGF stress. In addition, the polyamine transporter system was repressed as well, in which ATP-binding protein (VP1332), ABC transporter binding protein and permeases (VP1336–VP1338) were significantly down-regulated.

Based on the GSEA-GO analysis, a number of significantly changed biological functions were identified in SGP cells (*p* < 0.05) (Table 1), the majority of which were repressed in the AGF stress. Of these, the most down-regulated was a putative transporter (VPA1287) involved in Zn^2+^ transmembrane transport system. Likewise, the gene (VP2491) encoding a periplasmic iron-compound-binding protein in Fe^3+^ transport system was also significantly down-regulated. Low pH is thought to increase metal ion toxicity in bacteria, and an excess of metal ions causes oxidative damage [17]. Our data suggested perhaps decreased transport of the metal ions (Zn^2+^, Fe^3+^) into SGP cells after exposed to the AGF stress. In addition, three genes in the glycine betaine (GB) biosynthesis were down-regulated, which are involved in the conversions of choline to betaine aldehyde and betain aldehyde to GB. Among the most up-regulated biological functions in SGP cells was the pilus biosynthesis. The gene (VPA0725), encoding a putative TadB involved in Flp pili biogenesis [31], was up-regulated (3.04-fold) in the AGF stress, suggesting possibly enhanced biofilm formation to protect the bacterium from the detrimental acid stress.

Activation of phage-associated genes at low pH stress has been reported in *Lactobacillus reuteri* [20]. Strikingly, in this study, three genes (VPA1173–1175) encoding phage major capsid protein, phage capsid scaffolding protein and putative bacteriophage protein showed unusual expression features between the two growth-phases cells of *V. parahaemolyticus* CHN25 in the AGF stress. They were induced with a minor increase of 2.06–2.19-fold in LGP cells, but highly down-regulated in SGP cells, particularly the capsid-related genes showing strongly 40-fold reduced expression. In addition, expression of the bacteriophage Mu tail sheath protein (GpL, VP2725) was slightly repressed at both growth phases. It will be interesting to elucidate biological significance of the differently expressed phage-associated genes in the AGF stress in future studies.

### 2.6. Regulators Involved in the Response of V. parahaemolyticus CHN25 to the AGF Stress

The genome-wide transcriptome data also revealed a total of sixty-nine and nine changed regulators in *V. parahaemolyticus* CHN25 cells at LGP and SGP in the AGF stress, respectively (Table S2). They globally or specifically regulate a wide variety of cellular processes including environmental stresses in bacteria, such as DNA-binding transcriptional or response regulators; LysR-type transcriptional regulators (LTTRs); AraC/XylS-, AsnC-, LacI-, LuxR-, MarR- and TetR-family of regulators; and some other regulators involved in multiple metabolism pathways. Of these, the majority were down-regulated in LGP cells, whereas opposite expression characteristics were observed in SGP cells in the AGF stress.

Interestingly, several differentially expressed regulators were identified from the cells at the two growth phases, suggesting the growth phase-independent and AGF-dependent regulation in *V. parahaemolyticus* CHN25. Of these, the genes encoding a LacI-family transcriptional regulator (VP2393), repressing a *lac* operon in *E. coli* [32], and a putative transcriptional regulator (VPA0593) showed higher expression levels in the AGF stress. In contrast, expression of a regulator BetI (VPA1114) was repressed, which negatively regulated the *betT* and *betIBA* genes that govern GB biosynthesis from choline in response to choline in *E. coli* [33]. The repressed BetI perhaps in turn acivated the target genes in the AGF stress, which perhaps led to increased amount of GB to maintain the integrity of cell membranes against the damaging effects of the AGF stress, as in other stress responses to excessive salt, cold, heat and freezing in bacteria [34]. The possible link between the acid stress and GB, an osmoprotectant in osmotic stress, has also been reported in *Streptococcus pneumoniae* [16]. The molecular responses of bacteria to external environment signals are complex, but in which the two-component transduction systems have been known to play an important role [35]. Consistent with previous studies, a response regulator (VPA0737) belonging to the two-component signaling systems, which enable bacteria to sense, respond, and adapt to a wide range of environments, stressors, and growth conditions [35], was elicited by the AGF stress in *V. parahaemolyticus* CHN25. However, distinct responses of the regulator were detected, which was up- and down-regulated in expression in LGP and SGP cells, respectively, implying different regulatory strategies adopted by the bacterium for dealing with the same stressor between the two growth phases. In addition, interestingly, two AsnC-family transcriptional regulators (VPA1717, VPA0091), known as feast/famine regulatory proteins specifically involved in multiple cellular metabolisms in bacteria, displayed 5.0- and 3.5-fold increased expression in LGP and SGP cells, respectively, suggesting possible regulation functions in the AGF stress as those in the feast/famine stress [36].

Among the differentially expressed regulators in LGP cells, the LTTRs were the most abundant in the AGF stress, except putative regulators with currently unknown regulatory functions in the public databases. The LTTRs represent the most abundant type of globally transcriptional regulators in bacteria, which are involved in a wide range of cellular processes, e.g., cell division, quorum sensing, oxidative stress, virulence, motility, attachment and secretion [37]. In this study, a total of ten LTTRs were identified in LGP cells in the AGF stress, however, all of which were significantly repressed in expression, except the one (VP0067) with an opposite minor increase. Similarly, expression of several regulators were suppressed as well, all of which have been reported to directly regulate gene expression in response to environmental stimuli or coordinately regulate in a complex network in bacteria [38,39,40]. For example, an AraC/XylS-family regulator (VPA0531), one of the most common positive regulators in bacteria, showed a decrease of 5.26-fold in the AGF stress. Regulators belonging to this family have three major regulatory functions including stress responses to alkylating agents, antibiotics, organic solvents and heavy metals, as well as the transition from LGP to SGP [38]. In addition, approximately a dozen regulators controlling multiple metabolic pathways were also repressed in the AGF stress, e.g., the DNA-binding transcriptional regulators AraC, HexR and YidZ (VPA1678, VP1236 and VPA1575), the key components in bacterial gene regulatory networks that can sense fluctuations under internal and external conditions [41,42,43]. In contrast, 26.1% of the differentially expressed regulators in LGP cells displayed significantly enhanced expression in the AGF stress. Of these, the regulator (VP2866) belonging to the LuxR-family transcriptional regulators, which are key players in quorum sensing and coordinate gene expression in a variety of cellular functions [44], showed a higher expression level. Similarly, expression of an osmolarity response regulator OmpR (VP0154) involved in the EnvZ/OmpR signal transduction system was also enhanced in the AGF stress, which positively or negatively modulates multiple gene expression implicated in the control of *Y. enterocolitica* adaptation to high osmolarity, oxidative and low pH stresses [45].

For the SGP cells, expression of a regulator belonging to the LTTRs (VP1316) and a putative transcriptional regulator (VPA1689) were significantly increased, whereas expression of a phospoglycerate transport regulatry protein PgtC (VPA0825) and a putative transcriptional regulator (VP1154) were decreased in the AGF stress.

Taken together, the transcriptome data figured out a complex molecular regulatory network in *V. parahaemolyticus* CHN25 after exposed to the AGF stress, which lead to three major molecular snapshots. A number of regulators, acting as activators and or repressors of single or operonic genes or a series of regulatory cascades under different environmental stresses in bacteria, were elicited from LGP cells, which perhaps globally or specifically triggered cell responses to the AGF stress and controlled intracellular processes. In contrast, a considerable number of regulators remained unchanged in SGP cells under the same stress condition, which was consistent with the turndown feature at this growth phase. In addition, some growth-phase independent regulators were identified, which likely played crucial roles specifically in the AGF stress response in *V. parahaemolyticus* CHN25. Finally, the AGF stress appeared to mediate cross-talk regulation with some other environmental stimuli, e.g., osmotic and feast/famine stresses. An in-depth regulatory network in future studies will allow for better understanding of acid stress mechanisms in *V. parahaemolyticus*.

### 2.7. Possible Acid Stress Mechanisms in V. parahaemolyticus CHN25

In this study, expression of the genes directly or indirectly associated with the pumping out of protons (e.g., F1F0-ATPase) was significantly enhanced in LGP cells after exposed to a sub-lethal acid condition (pH 4.9). Moreover, two genes (VP2125 and VP2718) encoding Na^+^/H^+^ antiporters were also up-regulated, which are important not only for energy transduction, but also for intracellular pH regulation, extrusion of toxic Li^+^ (and Na^+^) and cell volume regulation in bacteria [46].

Production of ammonia has been known to be one of the major mechanisms in acid stress response in bacteria. In this study, a number of up-regulated genes involved in nitrogen metabolism (e.g., NrfBD, NrfAH, a glutamate dehydrogenase) were identified, which likely increased intracellular ammonia in LGP cells in the AGF stress. Moreover, expression of the *aspA* (VP2863) and *hutH* (VP1273) genes encoding aspartate ammonia-lyase and histidine ammonia-lyase, as well as the gene (VPA0254) encoding l-serine dehydratase 1 that converts serine into ammonia and pyruvate were also significantly increased. In contrast, alanine dehydrogenase (VP1103) and d-amino acid dehydrogenase small subunit (VP0623) showed down-regulated expression. In addition, the *nagE* (VPA0038) gene encoding a glucosamine-6-phosphate deaminase that converts fructose-6-phpsphate to glucN-phosphate were up-regulated in the AGF stress. It has been reported that urease located on bacterial cell surface may create a neutral microenvironment by hydrolysis of urea to carbon dioxide and ammonia [47]. Unexpectedly, no urease-related genes were identified in *V. parahaemolyticus* CHN25 in the AGF stress. Overall, these data may have supposed a strong link between the enhanced ammonia production via multiple metabolic pathways in LGP cells and the acid stress imposed on the bacterium.

One interesting observation from the transcriptome data was that the regulator AphB (VP2184) belonging to the LTTRs was not significantly elicited by the AGF stress. Moreover, unexpectedly, expression of the *cadAB* operon (VP2890–VP2891) involved in the proton-consuming lysine-decarboxylation pathway was strikingly down-regulated (20–25-fold) in LGP cells. This finding was inconsistent with previous studies (e.g., [12,48,49]). We questioned whether the saline concentration of the AGF resulted in the distinct observation, since it has been reported that *V. parahaemolyticus* RIMD2210633 grown in Luria-Bertani (LB) supplemented with 3% NaCl induced a stronger *cadA* response after acidification than cells grown in LB with 1% NaCl [50]. To address the interesting result, we treated *V. parahaemolyticus* CHN25 grown to LGP with the AGF supplemented with 3% NaCl instead of 0.21% NaCl, and then determined the *cadAB* gene expression by qRT-PCR analysis. The resulting data revealed that both *cadA* and *cadB* genes were highly up-regulated in mRNA levels (data not shown). Our data, coupled with the previous results, demonstrated that environmental saline concentration likely mediated an important cross-regulation in acid stress response in *V. parahaemolyticus*.

In addition, consistent with previous research, the *toxS* (VP0819) gene was significantly up-regulated in the AGF stress, which belongs to the ToxR-ToxS signal transduction system required for the acid stress response in *V. cholerae* [11]. In addition, our transcriptome data also revealed some other possible mechanisms in *V. parahaemolyticus* CHN25, such as the attenuated consumption of ammonia (e.g., VP0483) and enhanced production of HCO_3_^−^ (e.g., VP2514), to maintain intracellular pH homeostasis in the AGF stress.

## 3. Experimental Section

### 3.1. Bacterial Growth Conditions

*V. parahaemolyticus* CHN25 bearing a SXT/R391-like integrative and conjugative element has recently been characterized by Song *et al.* [15]. The bacterium was detected positive for the *tlh* gene, but featured no toxic *tdh* and *trh* genes. *V. parahaemolyticus* CHN25 was streaked from a frozen stock at −80 °C in our laboratory onto LB solid medium [51] adjusted to pH 8.5, 3% NaCl, and incubated at 37 °C overnight. One colony was then inoculated into 5 mL TSB liquid medium (pH 8.5, 3% NaCl) (Beijing Land Bridge Technology Co., Ltd., Beijing, China), and aerobically cultured at 37 °C with shaking at 175 rpm. The overnight culture was diluted 1:100 (*v*/*v*) into fresh TSB liquid medium adjusted to the pH range of 1.5–12.5 with 1 M HCl or 6 M NaOH, respectively, and incubated at 37 °C for 16–20 h. The growth curves were determined using a BioScreener (BioScreen, Helsinki, Finland).

### 3.2. AGF Survival Assay

The AGF survival assay was performed according to the method described previously [19] with slight modifications. Briefly, *V. parahaemolyticus* CHN25 was incubated in TSB liquid medium at 37 °C to LGP and SGP, defined as an optical density at 600 nm (OD_600 nm_) of 0.7 and 1.3, respectively. An aliquot of the bacterial culture (1 mL) was centrifuged at 3500 rpm for 2 min, and the cell pellet was resuspended with 1 mL of 1× AGF, containing 8.3 g proteose peptone, 3.5 g d-glucose, 2.05 g NaCl, 0.6 g KH_2_PO_4_, 0.147 g CaCl_2_, and 0.37 g KCl per/L [19]. The cell suspension was added into 4 mL of 1× AGF, and the acid-exposed cells were incubated at 37 °C for 0–60 min or 0–2 h for LGP and SGP cells, respectively. Culturable bacterial cells were enumerated at different time points via plating appropriate dilutions of cell culture onto LB solid medium. 

### 3.3. RNA Extraction and Microarray Analysis

Total RNA preparation was performed using RNeasy Protect Bacterial Mini Kit (QIAGEN Biotech Co., Ltd., Hilden, Germany) according to the manufacturer’s instructions. The DNA was removed from the samples using RNase-Free DNase Set (QIAGEN, Hilden, Germany), and its quality and quantity was assessed using the Agilent Bioanalyzer 2100 system (Agilent Technologies, Santa Clara, CA, USA). Two independently prepared RNA samples were used in each microarray experiment [52].

Microarray chip design, cRNA labeling, hybridization, scanning and analyses were conducted at Shanghai Biotechnology Co., Ltd. (Shanghai, China). An array of 15,000 specific 60-m oligonucleotides was designed based on predicted coding sequences from *V. parahaemolyticus* RIMD2210633 and *V. parahaemolyticus* CHN25, respectively. It contained 4711 probes and covered 99.72% of the genes in *V. parahaemolyticus* CHN25. A sample of 2 μg RNA was used to synthesize cDNA, which was further transcribed into cRNA using a transcription mix containing aa-UTP and T7 RNA polymerase. Cyanine-3 (Cy3) labeled cRNA was performed by Low Input Quick Amp Labeling Kit, One-Color (Agilent), followed by purification using RNeasy mini kit (QIAGEN), according to the manufacturer’s instructions. Each slide was hybridized with 600 ng Cy3-labeled cRNA using Gene Expression Hybridization Kit (Agilent) in an Agilent Microarray Hybridization Chamber (Agilent) at 65 °C. After 17 h hybridization, slides were washed with Gene Expression Wash Buffer Kit (Agilent) following the manufacturer’s instructions. Microarrays were scanned using Agilent Microarray Scanner (Agilent) and the data were extracted with Feature Extraction software version 10.7 (Agilent). Raw data were normalized by Quantile algorithm, Gene Spring software version 11.0 (Agilent). The average coefficient of variation (CV) was <0.15 as recommended by Agilent for the quality control. Normalized expression ratios were calculated for each gene and tested for significance with the criteria |fold change| > 2.0 and *p* < 0.05. The GSEA of differently expressed genes was supported by the eBioservice (http://sas.ebioservice.com/portal/root/molnet_shbh/index.jsp) (Shanghai Biotechnology Co., Ltd., Shanghai, China) against the GO (http://geneontology.org/) and KEGG (http://www.genome.jp/kegg/) database, respectively.

### 3.4. qRT-PCR Analysis

Selected differentially expressed genes and/or significantly enriched genes in microarray chip analysis were validated by qRT-PCR. Oligonucleotide primers were synthesized by Shanghai Sangon Biological Engineering Technology Services Co., Ltd. (Shanghai, China). The reverse transcription reaction was performed using PrimeScript RT reagent Kit With gDNA Eraser (Perfect Real Time) (Japan TaKaRa BIO, Dalian Company, Dalian, China) according to the manfacturer’s protocol. A 20 μL reaction volume contained 10 μL FastStart Universal SYBR Green Master (ROX), 5 μM each of the oligonucleotide primers, 2 μL template cDNA and appropriate volume of sterile ddH_2_O (Roche, Basel, Switzerland). All qRT-PCR reactions were performed in a 7500 Fast Real-Time PCR System (Applied Biosystems, Foster City, CA, USA) under the following conditions: initial denaturation at 95 °C for 10 min, followed by 40 cycles of denaturation at 95 °C for 15 s, and primer annealing at 60 °C for 60 s, according to the manufacturer’s instructions. The *pvuA* gene was used as the qRT-PCR reference gene as previously described [53,54]. Expression level of the *pvuA* gene in *V. parahemolyticus* CHN25 grown in TSB to LGP and SGP was used as a reference/baseline, respectively. The data were analyzed using the Applied Biosystems 7500 software, and the relative expression ratio was calculated for each gene by using the delta-delta threshold cycle (*C*_t_) method [55].

### 3.5. Microarray Data Accession Number

The microarray data have been deposited in the NCBI Gene Expression Omnibus (http://www.ncbi.nlm.nih.gov/geo/) under the accession number GSE63167.

## 4. Conclusions

This study constitutes the first investigation of *Vibrio parahaemolyticus* CHN25 response to the AGF under a sub-lethal acid condition using genome-wide microarray analysis. The transcriptome data revealed global-level distinct gene expression profiles of the bacterium with considerable variation over growth phases after exposed to the AGF (pH 4.9) for 30 min. *Vibrio parahaemolyticus* seemed to have evolved a number of molecular strategies for coping with the acid stress in a complex gene regulation network. Our data in this study will highly facilitate the in-depth research of environmental stresses and pathogenicity of the leading seafood-borne pathogen worldwide.

## Supplementary Materials

Supplementary tables can be found at http://www.mdpi.com/1422-0067/15/12/22539/s1.
